# Algorithmic Error Correction of Impedance Measuring Sensors

**DOI:** 10.3390/s91210341

**Published:** 2009-12-21

**Authors:** Oleg Starostenko, Vicente Alarcon-Aquino, Wilmar Hernandez, Oleg Sergiyenko, Vira Tyrsa

**Affiliations:** 1 Research Center CENTIA, Department of Computing, Electronics and Mechatronics, Universidad de las Américas, Puebla, 72820, México; E-Mail: vicente.alarcon@udlap.mx; 2 Department of Circuits and Systems in the EUIT de Telecomunicación, Universidad Politécnica de Madrid, Campus Sur UPM, Ctra. Valencia km 7, 28031, Madrid, Spain; E-Mail: whernan@ics.upm.es; 3 Engineering Institute, Autonomous University of Baja California, Blvd. Benito Juárez y Calle de la Normal S/N, col. Insurgentes Este, 21280, Mexicali, Baja California, México; E-Mail: srgnk@iing.mxl.uabc.mx; 4 Universidad Politécnica de Baja California, Calle de la Claridad S/N, Col Plutarco Elías Calles, 21376, Mexicali, Baja California, México; E-Mail: vera@upbc.edu.mx

**Keywords:** impedance measuring sensor, error correction, *C-V*, *G-V* characteristic meter

## Abstract

This paper describes novel design concepts and some advanced techniques proposed for increasing the accuracy of low cost impedance measuring devices without reduction of operational speed. The proposed structural method for algorithmic error correction and iterating correction method provide linearization of transfer functions of the measuring sensor and signal conditioning converter, which contribute the principal additive and relative measurement errors. Some measuring systems have been implemented in order to estimate in practice the performance of the proposed methods. Particularly, a measuring system for analysis of *C-V, G-V* characteristics has been designed and constructed. It has been tested during technological process control of charge-coupled device CCD manufacturing. The obtained results are discussed in order to define a reasonable range of applied methods, their utility, and performance.

## 1. Introduction

In spite of the strong efforts to develop and design high accuracy sensors these are devices which still contribute the most significant error to measurement results due to imperfections of the sensor transfer functions [1,2]. Usually the calibration or linearization of sensor transfer functions is achieved by compensation of sensor output signals by analog or digital components. Actually, there are a few proposals to solve the problem and some of them are integrated solutions which allow such electronic correction. However, these integrated circuits are usually expensive and/or are not suitable for general use because they have been designed for specific applications [3-5].

Actually, measuring devices and systems for analysis of complex parameters (impedance and admittance of electrical circuits on alternate current) are used in different applications due to their high speed (0.01–0.001 s) and low relative measurement error (0.01–0.1%) [2,6]. However, an increment of accuracy and speed is achieved by incorporating additional control circuits into measuring process increasing the cost of new devices [7]. To improve the metrological characteristics of measurement facilities without cost increments, error correction methods are used. The basic structure of such improved measuring systems usually includes a sensing element, a signal conditioning element, a signal processing element, and a data presentation element [1,2,8].

A sensing element is the most critical piece of any measuring system. The different physical nature of operations in a passive (modulating) or an active (self-generating) sensing element permits measurement of a wide variety of electrical and non-electrical values. For instance, impedance measurements are based on the proportional changing of electrical properties of sensing element, such as resistivity, permittivity, and permeability in function of input measured force, displacement, torque, pressure, thickness, temperature, speed, humidity, *etc.* The correct selection of sensing element and its mathematical model practically defines accuracy and resolution of any measurement system.

The signal conditioning element converts the output of the sensing element (with certain error) into a form suitable for further processing. Among the many methods for designing signal conditioning elements the most efficient for particular applications are the bridge iterating methods, the equal deflection methods, the network analysis or substitution methods for comparative measurements, high-speed direct conversion methods, the dynamic counting and resonant methods for capacitance and inductance measurements, and the oscillating methods for feedback systems [5,9,10].

The signal processing element is implemented using analog to digital converters (ADC) and microcomputers that are high-accuracy devices that do not reduce the precision of the measurements. Other important functions of the processing element are error reduction or compensation, control and synchronization of sensing and conditioning element, and data visualization.

There are some well-known methods used for error correction by signal processing elements. They may be subdivided in the groups of compensative methods (a measurement result is used as a feedback compensation action on sensing or conditioning elements); differential methods [a measured parameter presented by an active value (voltage or current) is compared with a highly-stable reference voltage or current computing deviation of a measured parameter with respect to a reference one]; direct conversion methods (the measured parameter is treated in a sequential process of corresponding active value conversion) [1,4,5,7,11]. These approaches linearize non-linear transfer functions of a sensor, forward channel, or a whole measuring system reducing steady-state errors and increasing sensitivity and resolution of the measuring device. Complex value measuring systems, due to their non-destructivity, are usually used for analysis of linear and non-linear electric circuits in alternating current, control of technological processes in semiconductor manufacturing, in low-frequency dielectric spectroscopy, bio-electrical impedance tomography, electrical impedance control, treatment of food, and in scientific research [8,9]. In order to improve the metrological characteristics of measuring systems without increasing cost and reducing operational speed, some novel error correction methods are proposed and implemented in this paper.

## 2. Selection of Measurement Method for Impedance Analysis

Any measured value can be represented by an equivalent circuit, which consists of serial and parallel connections of resistance, inductance, and capacitance. The equivalent or substitution circuit is varied according to the magnitude and frequency of the test exciting signal. Impedance measurements strongly depend on specific characteristics of the sensor and data acquisition process, such as limited power (25 mV) and imperfection of monochromatic sine test signals generating non-linear distortions, low magnitudes of the parameter to be measured (10^−3^−10^2^ pF for capacitance and 10^−4^−10 μS for conductance) requiring high resolution of the impedance meter, variable configurations of equivalent circuits in measuring converters due to wide test signal frequency range from 1 Hz to 1 MHz, *etc.*

**Figure 1.**
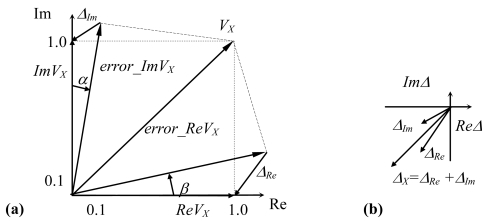
(a) Orthogonal components of the measured complex voltage *V_X_*. (b) Error vector due to parasitic rotations of orthogonal axes.

The total accuracy of measurement is defined by additive and multiplicative errors of measuring converters which may be represented as error vectors with corresponding active and reactive components, as shown in Figure 1. The complex measured voltage *V_X_* is interpreted as active *ReV_X_*, and reactive *ImV_X_* components which may be measured with real *Δ_Re_* and imaginary *Δ_Im_* components of absolute errors vector *Δ_X_* [8,10,12]. The error vector depends on orthogonal axes parasitic rotations with angles *α* and/or *β* [see Figure 1(b)]. The improvement of measuring equipment now consists in estimation and reduction or compensation of systematic errors of the used method.

**Figure 2.**
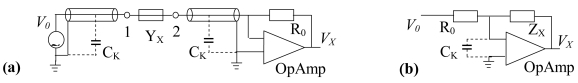
Electric circuit of normal active converters: (a) for complex conductance and (b) for complex resistance measurements

The measuring converter of any sensor is a critical unit of the meter because it introduces the biggest error. Usually passive or active measuring converters are used [9]. The passive converter is a simple low accuracy voltage divider, which consists of measured and test resistors. Chiefly, the operational amplifier (OpAmp) with negative feedback on voltage is used in active converters which are classified as conductance or resistance and normal or inverted converters. The block diagrams of some active converters are shown in Figure 2. Both the stray capacity and the input leakage conductance are always presented on the input of the measuring converter. For resistance-voltage converter the simplified circuit is presented in Figure 2b. In the following we will use only analysis of the normal conductance active converters, since the procedure for others is similar.

For ideal complex conductance conversion (*Y_X_*→*V_X_*) using an OpAmp, the output voltage, proportional to measured value, is calculated as:

(1) $$V_{X}=-Y_{X}R_{0}\frac{1}{1+\overset{1}{\;}/\underset{\beta A}{\;}}V_{0}\approx -Y_{X}R_{0}V_{0}$$

The feedback gain *βA* depends on the parameters of an OpAmp. The errors of test signal deviation and test resistor *R*_0_ instability may be excluded due to their small magnitude (about 0.001%) [9]. The errors of stray capacity and input leakage conductance of measuring converter, as well as the impedance of the input cable have additive character and can be removed by the initial zero correction of the measurement system. Therefore, we will evaluate only systematic errors of the measurement method when, for example, active conductance converters are used.

## 3. Proposed Error Correction Methods for Impedance Measurements

### 3.1. Structural Method of Algorithmic Error Correction

The structural method of error correction is based on insertion into the direct or feedback channel of measuring system of additional high-speed analog or digital units, which linearize the total non-linear transfer functions of the designed circuit [13,14]. We introduce an error correction method which combines the simplicity and speed of direct channel measurements and the high accuracy of feedback systems. For this, the output voltage and relative error vector of the active converter (see Figure 1) with a presence of input cable capacitance *C_K_* are calculated as:

(2) $$V_{X}=-Y_{X}R_{0}\frac{1}{1+i\;f/f_{T}\;\left[1+R_{0}\left(Y_{X}+i\omega C_{K}\right)\right]}V_{0};\;\delta =i\;f/f_{T}\;\frac{1+R_{0}\left(Y_{X}+i\omega C_{K}\right)}{1+i\;f/f_{T}\left[1+R_{0}\left(Y_{X}+i\omega C_{K}\right)\right]}$$

where *A*_0_ is the direct current gain of the OpAmp without feedback, *f_T_* is the frequency of unity amplification, *f* is the frequency of the test signal. For example, if *C_K_* = 100 nF, *ω* = *2πf* = 6,280 s^−1^, *Y_X_* = (1 + *i*0) μS, *f_T_* = 10^7^ Hz then |*δ*| ≈ 0.02%. The transfer function of the whole active conductance converter without frequency-range dependence may be presented as:

(3) $$G_{Y}=\frac{X_{1}+iX_{2}}{1+\left(K_{0}+\text{iMF}\right)\;\left(1+X_{1}+iX_{2}+\text{iTF}\right)}$$

where *Y*_1_, *Y*_2_ are the active and reactive components of the converted conductance *Y_X_, X*_1_ = *Y*_1_*R*_0_, *X*_2_ = *Y*_2_*R*_0_, *TF* = *2πfC_K_R*_0_, *K*_0_ = *1/A*_0_, *MF* = *f/f_T_*. The ideal transfer function is found now as *G_ideal_* = *X*_1_ + *iX*_2_ and with *G*_1_ = *Re(G_ideal_), G*_2_ = *Im(G_ideal_)*, the relative error vector of conversion for active and reactive components may be found as:

(4) $$\delta_{1}=G_{1}/X_{1}-1-=\varphi_{1}\left(X_{1},X_{2},TF,MK,K_{0}\right)\;\delta_{2}=G_{2}/X_{2}-1-=\varphi_{2}\left(X_{2},X_{1},TF,MK,K_{0}\right)$$

Thus, the analysis of errors is reduced to computing the functions *ϕ_1_* and *ϕ_2_*. On the basis of previous equations, active and reactive components of conversion errors are calculated (a detailed description of error analysis may be found in [8]):

(5) $$\begin{array}{c} \delta_{1}=\frac{-\left(1+X_{1}-X_{2}^{2}/X_{1}\right)K_{0}+\left(2+1/X_{1}\right)X_{2}MF+Q}{1+2\left(1+X_{1}\right)K_{0}-2X_{2}MF+Q}\\ \delta_{2}=\frac{-\left(1+2X_{1}\right)K_{0}+\left(X_{2}+X_{1}/X_{2}-X_{1}^{2}/X_{2}^{2}\right)MF+Q}{1+2\left(1+X_{1}\right)K_{0}-2X_{2}MF+Q};\;Q=\left[{\left(1+X_{1}\right)}^{2}+X_{2}^{2}\right]\left(K_{0}^{2}+MF^{2}\right) \end{array}$$

With the obtained Equations (5), the components of error vectors have been calculated for different combinations between *X*_1_ and *X*_2_. For example, if in the measured structure the active component predominates, then *X*_1_ = 1.0 and *X*_2_ = 0.1; otherwise, if the reactive component predominates, then *X*_1_ = 0.1 and *X_2_* = 1.0. Taking into account two non-linear equations for active and reactive components of conductance converters transfer function (Equation 3) and applying their linearization according to:

(6) $$X_{1}=G_{1}\left(1-\overset{\delta_{1}}{\;}/\underset{1+\delta_{1}}{\;}\right)\approx G_{1};\;X_{2}=G_{2}\left(1-\overset{\delta_{2}}{\;}/\underset{1+\delta_{2}}{\;}\right)\approx G_{2}$$

the approximate solution for the correction algorithm is defined as:

(7) $$\begin{array}{l} N_{1}=G_{1}+\left[\frac{G_{1}\left(1+G_{1}\right)}{A_{0}}-\frac{G_{2}\left(TF+G_{2}\right)}{A_{0}}-\frac{G_{1}\left(TF+G_{2}\right)}{f_{T}/f}-\frac{G_{2}\left(1+G_{1}\right)}{f_{T}/f}\right]\\ N_{2}=G_{2}+\left[\frac{G_{2}\left(1+G_{2}\right)}{A_{0}}-\frac{G_{1}\left(TF+G_{2}\right)}{A_{0}}-\frac{G_{2}\left(TF+G_{2}\right)}{f_{T}/f}-\frac{G_{1}\left(1+G_{1}\right)}{f_{T}/f}\right] \end{array}$$

In Equations (7) the components in brackets are correction actions which must be added to corresponding components of the transfer functions *G*_1_ and *G*_2_ in order to obtain accurate results. These equations may be simplified without significant reduction of precision due to *1/A_0_*≈*0*:

(8) $$N_{1}=G_{1}-\left[G_{1}\left(TF+G_{2}\right)+G_{2}\left(1+G_{1}\right)\right]MF;\;N_{1}=G_{2}-\left[G_{2}\left(TF+G_{2}\right)-G_{1}\left(1+G_{1}\right)\right]MF$$

Therefore, the improvement of metrological characteristics is achieved by computing components of the systematic error and its compensation by additional operations according to the described algorithm. We propose to name this technique the “structural algorithmic correction method”. The additional arithmetic operations in the Equation 8 are implemented using analog adders and multipliers based on OpAmp. Input variables such as *R*_0_ or *TF* are simulated by reference direct-voltage dividers and frequency-to-voltage converters, respectively [8].

**Figure 3.**
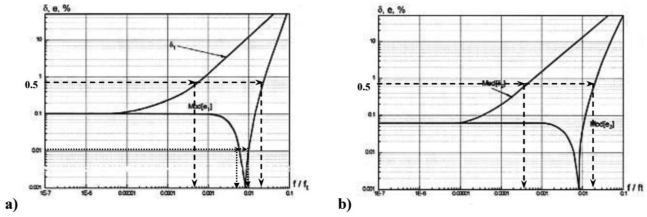
(a) Error behavior for active component of conductance measuring converter without (*δ*_1_) and with (*e*_1_) correction. (b) Error behavior for reactive component without (*δ*_2_) and with (*e*_2_) correction.

The plots for error behavior without the correction algorithm *δ*_1_ and *δ*_2_ according Equation (5) are shown in Figure 3 as the functions *δ*_1_=*ϕ*_1_*(f/f_T_)* and *δ*_2_=*ϕ*_2_*(f/f_T_)*. In the same plot the results with correction *e*_1_ and *e*_2_ computed according to expression (9) are shown for conductance measuring converters, where:

(9) $$e_{1}=\frac{N_{1}}{X_{1}-1},\;e_{2}=\frac{N_{2}}{X_{2}-1}$$

In a wide range of frequencies, the errors of the measuring converter sometimes change their sign, so the absolute values of errors *Mod*[*e*_1_], *Mod*[*e*_2_] are presented in Figure 3.

Analysis of errors has been made for cheap OpAmp with a small gain factor *A*_0_ = 20,000, the frequency of unitary amplification *f_T_* = 10 MHz, and test signal frequency range from *f* = 1 Hz to 1 MHz corresponding to *MF* = 10^−7^−10^−1^. The results of the experiments presented in Figure 3 show specific intervals of frequencies, where the error can be less than 0.001%. For example, in order to achieve an error of about 0.01%, the measurements must be done in the 40–100 kHz frequency range. This interval depends on the parameters of OpAmp, the magnitude of the reference resistor, and the frequency of the test signal. Therefore, the low error band may be computed and selected for a particular application! As result, it is possible to design cheap and high performance impedance meters with very low errors.

Another unexpected result of implementation of the structural algorithmic correction method is the measurement frequency range extension with the same level of errors. For example, a measurement error of 0.5% without correction (curves *δ*_1_ and *δ*_2_) can be achieved only in the frequency interval up to *f/f_T_* ≈ 0.0003/0.0004 (*f* ≈ 3/4 kHz). For conductance conversion with correction, the errors *e_1_* and *e_2_* have the same level up to the frequency interval *f/f_T_* ≈ 0.02 (*f* ≈ 200 kHz). This means that the error correction method reduces the error level and maintains it in a wide test signal frequency range. In the previous example with a conductance converter the frequency extension covers two orders of magnitude; this is significant improvement! Another advantage of the structural algorithmic correction method is that the measurements are performed without speed reduction (during the same measurement cycle). The propagation delay for analog correction circuits (in the worst case: hundreds of microseconds) does not have a significant effect over measurements in millisecond cycles. Moreover, the impedance meter is quite cheap due to the basic quality of electronic components used in measuring converters and correction unit. For example, as shown in Figure 3, errors as low as 0.001% have been achieved with OpAmps with a gain factor only about 20,000 (modern OpAmps have *A*_0_ gain factors of more than 10^6^).

The principal disadvantage of this method is that a correction unit can be a complex circuit because it must support many arithmetic operations for generation of corrective actions. This problem may be solved by standardization and unification of analog circuits for simple arithmetic operations in high-scale integrated circuits. There is no correlation between measured active (real) and reactive (imaginary) components of measured value as well as between the components of systematic errors that appear during impedance measuring processes. This is because the result basically depends on the physical characteristics of the measured value and not on the proportion between real and imaginary parts of measured value. If a measured value is a characteristic of semiconductor structure, the impedance measured components are better used for describing its features, such as substrate doping, flat band voltage, mobile ion concentration, series of resistance effects, charge densities, loss in the MOS structure due to charging and discharging of interface traps, *etc.*

The structural algorithmic correction method has been introduced for linearization of the transfer function of a complex conductance measuring converter. However it is only the first stage of the measurements. The consecutive vector-scalar and analog-to-digital converters introduce non-linear errors which must be removed by their own correction structures that may occupy more than 30% of all the measuring equipment. As usual, these structures are simple to implement and may be used for incrementing the performance of cheap measuring systems.

### 3.2. Iterating Correction Method

**Figure 4.**
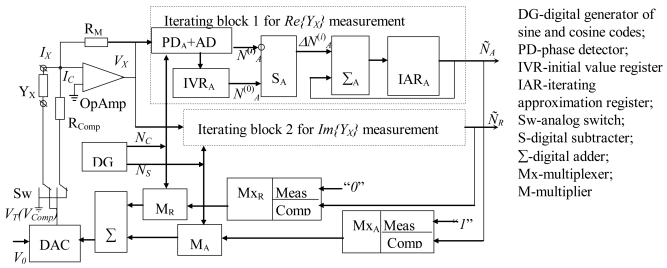
Block diagram of impedance meter with iterating error correction.

In order to explore the power of other error correction techniques an iterating compensation method is also proposed. Iterating methods use error correction units incorporated into the feedback channel which generates correction signals according to the results of previous measurement steps [15]. The functional block diagram of the proposed system is shown in Figure 4. The system consists of a digital generator (DG) of orthogonal signals, a measuring unit of varying configuration with OpAmp for direct measurement and iterating correction modes, a direct channel for measuring the active and reactive components with its corresponding ADCs, two iterating blocks for active and reactive components, and feedback units for the generation of compensation voltages. The measuring circuit formed by an active converter of conductance *Y_X_* generates a proportional complex current *I_X_* and then transfers it into a complex voltage *V_X_*. In other words, this circuit is a vector converter, which consists of an OpAmp with scale feedback resistor *R_M_*, compensation resistor *R_Comp_*, and switch Sw. DG forms orthogonal sine and cosine signals which are defined by two 10-bits codes for sine *N_S_* and cosine *N_C_* functions:

(10) $$N_{S}(kT)=\sin(2\pi \text{kTf});\;N_{C}(kT)=\cos(2\pi \text{kTf})$$

where *k* is the number of clock pulses, and *f* is test signal frequency. These codes are used as reference signals for phase detectors (PD), for generation of analog test (*V_T_*) and compensation (*V_Comp_*) voltages. The measurement process is divided into two steps: impedance measurement without correction and iterative correction of the result. First, the sine *V_T_* voltage is generated at the output of DAC by a look-ahead digital adder Σ. For this, the multiplier M_A_ repeats the sine codes *N_S_*(*kT*) on its output because the set of ones “*1*” from measurement mode “*Meas*” of M_A_ is selected. These ones repeat the codes *N_S_* (sine signal) in the output of M_A_ in measurement mode. The cosine signal codes are multiplied by zero from input “*Meas*” of M_R_. Therefore, the *V_T_* is computed as *V_T_*(*kT*)=*bV*_0_cos(2*πkTf*) where *b* is the transfer coefficient and *V_0_* is the reference voltage of DAC. *V_T_* is applied to the measured impedance *Y_X_* through switch Sw. The output voltage *V_X_* and transfer function *G_VC_* of vector converter are obtained as:

(11) $$V_{X}=I_{X}G_{VC}=\left(Y_{A}+iY_{R}\right)G_{VC}V_{T};\;G_{VC}=\frac{R_{M}}{1+i\overset{f}{\;}/\underset{f_{T}}{\;}\;\left[1+R_{M}\left(Y_{A}+iY_{R}+\overset{1}{\;}/\underset{R_{K}}{\;}+i2\pi fC_{K}\right)\right]}$$

where *Y_A_* and *Y_R_* are active and reactive components of measured value *Y_X_, f_T_* is the frequency of unitary amplification, and *C_K_* is capacitance of the input cable.

The phase sensitive detectors PD_A_, PD_R_ convert *V_X_* into active and reactive components by comparing them with the signals of DG in phase and with a 90° phase shift (sine and cosine signals codes). This conversion is described by equations:

(12) VA=c[Re{VX}cos{ψA}−Im{VX}sin{ψA}];ψA=2πf(tD+2Im{VX}/S);VR=c[Im{VX}cos{ψR}+Re{VX}sin{ψR}];ψR=2πf(tD+2Re{VX}/S)

where *c* is the transfer coefficient of the phase detector, *ψ_A_* and *ψ_R_* are parasitic phase shifts during the selection of active and reactive components (in Figure 1 they are the angles *α* and *β*), *t_D_* is internal comparator delays, *S* is the speed of increasing the output voltage of OpAmp.

The digital codes of the measured components are obtained by ADC_A_ and ADC_R_ with their nominal transfer coefficient *q_N_*, and absolute additive *Δ_Ad_* and relative multiplicative *δ_Mul_* errors:

(13) $$N_{A}^{\left(0\right)}=q_{N}\left(1+\delta_{\text{Mul}}\right)V_{A}+\Delta_{Ad};\;N_{A}^{\left(0\right)}=q_{N}\left(1+\delta_{\text{Mul}}\right)V_{R}+\Delta_{Ad}$$

The magnitudes of components *N_A_* and *N_R_* are stored into the initial values registers IVR_A_, IVR_R_ and into the iterating approximation registers IAR_A_, IAR_R_ for iterating compensation cycles. These codes may be used as final results of the measurements. If higher accuracy is required of the results, the iterating process is applied. The total errors of first impedance measurement components are computed according to (13) as:

(14) $$N_{A}^{\left(0\right)}=b\alpha_{N}\left(1+\delta_{A}^{\left(0\right)}\right)Y_{A};\;N_{R}^{\left(0\right)}=b\alpha_{N}\left(1+\delta_{R}^{\left(0\right)}\right)Y_{R}$$

where *α_N_* is the nominal coefficient of direct channel transfer. In Table 1, the relative errors *δ^(0)^_A_, δ^(0)^_R_* of conductance *Y_X_* = (100 + *i*100) *S* measurements are shown, *R_K_* = 1 kΩ, *f_T_* = 10 MHz, *S* = 50 V/mks, *t_D_* = 10 *ns*, scale-resistor non-stability *δ_M_* = 5%, *Δ_A_* = 0.5 units of the least significant digit.

Iterating error correction begins with the generation of the first iterating compensation voltage *V*^(1)^*_Comp_* based on the codes *N*^(0)^*_A_* and *N^(^*^0)^*_R_* from IAR_A_, IAR_R_. After the modulation of these codes by DG-codes producing *N_C_(kT)* and *N_S_(kT)* the magnitude of the first compensation voltage is found as:

(15) $$V_{\mathrm{Comp}}^{\left(1\right)}(kT)=bV_{0}\left[{\tilde{N}}_{A}^{\left(1\right)}\cos\left(2\pi Tf\right)+{\tilde{N}}_{R}^{\left(1\right)}\sin\left(2\pi Tf\right)\right]$$

The voltage from Equation (15) through the normally closed contacts of Sw is applied to the compensation resistor R_Comp_. The output voltage *V^(1)^_X_* of measuring converter is appears as:

(16) $$V_{X}^{\left(1\right)}=I_{X}^{\left(1\right)}G_{VC}=\frac{V_{\mathrm{Comp}}}{R_{\mathrm{Comp}}}G_{VC}$$

The first iterating approximation result of *V^(1)^_X_* is found after analog-to-digital conversion as:

(17) $$N_{A}^{\left(1\right)}=q_{N}\left(1+\delta_{\text{Mul}}\right)V_{A}^{\left(1\right)}+\Delta_{Ad};\;N_{R}^{\left(1\right)}=q_{N}\left(1+\delta_{\text{Mul}}\right)V_{R}^{\left(1\right)}+\Delta_{Ad}$$

The feedback iterating blocks, which consist of IVRs, IARs, subtractors S_A_, S_R_, and adders Σ_A_, Σ_R_ generate the correction signals: $\Delta N_{A}^{\left(1\right)}=N_{A}^{\left(0\right)}-N_{A}^{\left(1\right)};\;\Delta N_{R}^{\left(1\right)}=N_{R}^{\left(0\right)}-N_{R}^{\left(1\right)}$ which then are added to previous results of measurements from IAR registers producing:

(18) $${\tilde{N}}_{A}^{\left(1\right)}={\tilde{N}}_{A}^{\left(1\right)}+\Delta {\tilde{N}}_{A}^{\left(1\right)};\;{\tilde{N}}_{R}^{\left(1\right)}={\tilde{N}}_{R}^{\left(1\right)}+\Delta {\tilde{N}}_{R}^{\left(1\right)}$$

The iterating-measuring process is continued according to the described algorithm (Equations 15–18). In general form, the iterating correction values of the *(n* + *1)-th* approximation are found as:

(19) $${\tilde{N}}_{A}^{\left(n+1\right)}={\tilde{N}}_{A}^{\left(n\right)}+\left({\tilde{N}}_{A}^{\left(0\right)}-{\tilde{N}}_{A}^{\left(n\right)}\right);\;{\tilde{N}}_{R}^{\left(n+1\right)}={\tilde{N}}_{R}^{\left(n\right)}+\left({\tilde{N}}_{R}^{\left(0\right)}-{\tilde{N}}_{R}^{\left(n\right)}\right)$$

According to the convergence condition of the iterating correction algorithm, the increment of approximation numbers leads to the fact that *N^(i)^_A_, N^(i)^_R_* approach to *N^(0)^_A_, N^(0)^_R_*. At the step *I* = *m* when the conditions $N_{A}^{\left(m\right)}=N_{A}^{\left(0\right)}$ and $N_{R}^{\left(m\right)}=N_{R}^{\left(0\right)}$ are satisfied with an accuracy about the least significant digit of the compared codes the correction is finished and the values:

(20) $$N_{A}^{\left(m\right)}=\frac{\beta \alpha_{N}}{R_{\mathrm{Comp}}}\left(1+\delta_{A}^{\left(m\right)}\right){\tilde{N}}_{A}^{\left(m\right)};\;N_{R}^{\left(m\right)}=\frac{\beta \alpha_{N}}{R_{\mathrm{Comp}}}\left(1+\delta_{R}^{\left(m\right)}\right){\tilde{N}}_{R}^{\left(m\right)}$$

give the accurate results of the iterating impedance measurements. The *δ^(m)^_A_* and *δ^(m)^_R_* are the relative errors of the conversion at step *m*. The accuracy limit of measurement depends on the precision of the compensation resistor, time instability of direct and feedback channels, and non-linearity of the DAC.

Table 2 shows the relative errors during the iterating correction process for measurements on a test signal frequency equal to 500 kHz. Table 3 presents the results of experiments for the determination of necessary steps to achieve measurement errors of less than ±0.05% for a wide range of frequencies

The advantage of the proposed iterating method consists in the generation of correction actions in feedback channels in digital form. That results in a very small error equal to half of the least significant bit of the operating word of the DAC (±0.5/2^n^ where *n* is the number of input bits of DAC). The iterating correction may be used for accurate measurements up to 500 kHz (Table 3) due to the increment of required iterations as a result of the tight interaction of converter channels at high frequencies.

## 4. Practical Implementation of Error Correction Methods

The experimental results concerning the efficiency of the proposed methods were obtained using an automatic parameter control system which has been constructed for technological process control of charge-coupled device (CCD) manufacturing. The parameter control is used to estimate the accuracy of manufacturing conditions in order to detect deviation limits of the electrical and physical characteristics of the output product. Usually multi-frequency Volt-Farad, Volt-Siemens *C-V, G-V* plots are used for characterization of semiconductor structure quality, providing all the necessary information for correcting electrical characteristics of samples in real-time [16].

Additionally to *C-V, G-V*-characteristic measurements (capacitance or conductance of the semiconductor is a function of direct current displacement) the capacitance relaxation *C-t*-measurements are used where impedance of structure is analyzed after its excitation by pulsed voltage. The latest instruments from Hewlett-Packard, Keithley, Boonton, and Materials Development Corporation show the importance of advanced research and development of measuring tool using *C-V, G-V* characteristics in diverse applications, such as ion implant analysis of thin film transistors, MOS capacitance-time measurements, typical four-terminal point-contact spectroscopy by phase-related measurements, *etc.* [17,18].

**Figure 5.**
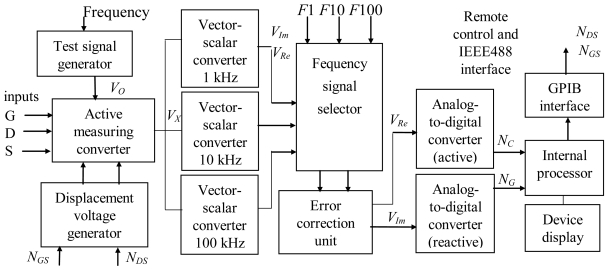
Block diagram of the measurement system for automatic control of semiconductor characteristics.

The automatic parameter control system for semiconductor manufacturing test has been designed and constructed applying the direct capacitance measurement method with algorithmic error correction of real *V_Re_* and imaginary *V_Im_* components. This approach provides high-speed measurement and precise results that satisfy all the requirements of a semiconductor parameter analysis. The block diagram of *C-V, G-V* characteristic measurement system is presented in Figure 5. There are two operating modes in the proposed measurement system: the first provides single parameter measurement, when only *C_X_* and *G_X_* values are measured at any selected frequency. The second mode supports automatic measurement of the complete *C-V, G-V* characteristics and transfers the data to external computer connected through the standard high-speed GPIB (IEEE488) interface [19].

The measuring process is started by the connection of a measured structure to three terminals (G, S, D) and selection of measuring ranges, frequencies of test signal, and displacement voltages *V_GS_, V_DS_* (±40 V). It is possible for higher system robustness to implement additionally the test signal frequencies feedback control under the novel original method introduced in [20-22]. The measured complex value *V_X_* is converted by a vector-scalar converter into real and imaginary components (*V_Re_, V_Im_*) which are then represented by digital codes. In the single parameter measurement mode the results, test and displacement voltages, frequency, and range of measurements are displayed on a device display. The error correction unit in this system is used only for linearization of the phase detector transfer function, because for parameter control of semiconductor manufacturing process a 0.1–1% error range is acceptable. For this reason, simplified measuring channels may be designed.

In this case, the transfer function of phase detector is analyzed according to the previously proposed error correction algorithm. The principal errors arise from deviations of parasitic angles *α* and *β* (see Figure 1) and due to tight interaction between channels for active and reactive component conversion. These errors are removed by the correction unit based on a set of adders and multipliers.

**Figure 6.**
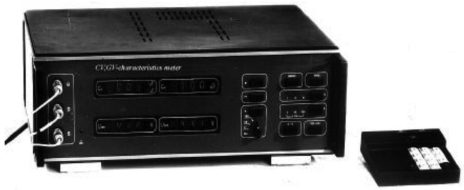
*C-V, G-V* meter for control of semiconductor characteristics.

To extend the operational functionality of a system the remote control unit (external keyboard) can be included. The constructed measurement system is represented in Figure 6 and its metrological characteristics are shown in Table 4. In the Table *C_X_* and *G_X_* are the results of capacitance and conductance measurements, *C_1N_* and *G_1N_* are the maximum nominal values for selected range. The measured limits are divided into three intervals, such as 0.1 pF/1 nS, 1 pF/0.01 μS, and 0.01 nF/100 μS.

## 5. Conclusions

Two error correction methods based on linearization of transfer functions of impedance measuring system have been proposed and tested. These methods require simple and cheap electronic components and provide high accuracy measurements without reduction of conversion and data processing speed. Based on the direct conversion method a measurement system for automatic control of semiconductor characteristics during the manufacturing process has been designed and constructed. The system provides the measurement of the *C-V, G-V* characteristics of semiconductor structures over a wide range of test signal frequencies and displacement voltages. The principal disadvantage of the proposed approaches is that a correction module may be a complex circuit due to utilization of a great number of units that implement the arithmetic operations needed for generating the corrective actions. This problem may be solved by standardization and unification of analog circuits in specialized high-scale integrated circuits, which may be used for execution of simple arithmetic operations in analog form.

## Figures and Tables

**Table 1.** Relative errors of measurement of active and reactive components without correction.

**kHz**	1	5	10	20	50	100	200	500	1000
**δ^(0)^_A_,%**	-1.96	-1.78	-1.55	-1.09	0.31	2.77	8.06	26.7	64.4
**δ^(0)^_R_,%**	-2.05	-2.33	-2.66	-3.3	-5.22	-8.33	-14.2	-29.4	-46.7

**Table 2.** Relative errors of measurement on *f* = 500 kHz with iterating correction.

**Iter.(*m*)**	0	1	2	3	4	5	6	7	8
***δ_A_*^(*M*)^**	24	−16	−1.9	2.5	0.07	−0.38	0.02	0.05	0.01
***δ_R_*^(*M*)^**	−34	−5.7	5.9	−0.38	−0.89	0.02	0.13	−0.01	0.00

**Table 3.** Number of iterating steps for impedance measurement with relative error 0.05%.

***f*, kHz**	1	2	5	10	20	50	100	200	500	1000
**steps**	2	2	2	2	2	2	3	4	7	22

**Table 4.** Parameters, measurement intervals, and errors of *C-V, G-V* characteristic measurement system.

**Parameter, measurement ranges**		**Measurement Errors at 1 kHz, (%)**	**Measurement Errors at 10 kHz, (%)**	**Measurement Errors at 100 kHz, (%)**
C_X_	1	$\pm \left[0.5+0.2\left(\frac{C_{1N}}{C_{X}}-1\right)+0.2\frac{G_{X}}{C_{X}}\right]$	$\pm \left[0.5+0.2\left(\frac{C_{1N}}{C_{X}}-1\right)+0.2\frac{G_{X}}{C_{X}}\right]$	$\pm \left[0.5+0.2\left(\frac{C_{1N}}{C_{X}}-1\right)+0.2\frac{G_{X}}{C_{X}}\right]$
C_X_	2	$\pm \left[0.2+0.1\left(\frac{C_{1N}}{C_{X}}-1\right)+0.2\frac{G_{X}}{C_{X}}\right]$	$\pm \left[0.2+0.2\left(\frac{C_{1N}}{C_{X}}-1\right)+0.2\frac{G_{X}}{C_{X}}\right]$	$\pm \left[0.5+0.2\left(\frac{C_{1N}}{C_{X}}-1\right)+0.2\frac{G_{X}}{C_{X}}\right]$
C_X_	3	$\pm \left[0.5+0.2\left(\frac{C_{1N}}{C_{X}}-1\right)+0.2\frac{G_{X}}{C_{X}}\right]$	$\pm \left[0.5+0.2\left(\frac{C_{1N}}{C_{X}}-1\right)+0.2\frac{G_{X}}{C_{X}}\right]$	$\pm \left[0.5+0.2\left(\frac{C_{1N}}{C_{X}}-1\right)+0.2\frac{G_{X}}{C_{X}}\right]$
G_X_	1	$\pm \left[0.2+0.1\left(\frac{G_{1N}}{G_{X}}-1\right)+0.1\frac{C_{X}}{G_{X}}\right]$	$\pm \left[0.2+0.1\left(\frac{G_{1N}}{G_{X}}-1\right)+0.1\frac{C_{X}}{G_{X}}\right]$	$\pm \left[0.5+0.2\left(\frac{C_{1N}}{C_{X}}-1\right)+0.1\frac{G_{X}}{C_{X}}\right]$
G_X_	2	$\pm \left[0.2+0.1\left(\frac{G_{1N}}{G_{X}}-1\right)+0.1\frac{C_{X}}{G_{X}}\right]$	$\pm \left[0.2+0.1\left(\frac{G_{1N}}{G_{X}}-1\right)+0.1\frac{C_{X}}{G_{X}}\right]$	$\pm \left[0.5+0.2\left(\frac{G_{1N}}{G_{X}}-1\right)+0.1\frac{C_{X}}{G_{X}}\right]$
G_X_	3	$\pm \left[0.5+0.2\left(\frac{G_{1N}}{G_{X}}-1\right)+0.1\frac{C_{X}}{G_{X}}\right]$	$\pm \left[0.5+0.2\left(\frac{G_{1N}}{G_{X}}-1\right)+0.2\frac{C_{X}}{G_{X}}\right]$	$\pm \left[1.0+0.2\left(\frac{G_{1N}}{G_{X}}-1\right)+0.2\frac{C_{X}}{G_{X}}\right]$
